# mTOR Senses Environmental Cues to Shape the Fibroblast-like Synoviocyte Response to Inflammation

**DOI:** 10.1016/j.celrep.2018.04.044

**Published:** 2018-05-15

**Authors:** Thomas Karonitsch, Richard K. Kandasamy, Felix Kartnig, Barbara Herdy, Karolina Dalwigk, Birgit Niederreiter, Johannes Holinka, Florian Sevelda, Reinhard Windhager, Martin Bilban, Thomas Weichhart, Marcus Säemann, Thomas Pap, Günter Steiner, Josef S. Smolen, Hans P. Kiener, Giulio Superti-Furga

**Affiliations:** 1 Division of Rheumatology, Department of Medicine 3, Medical University of Vienna, 1090 Vienna, Austria; 2 CeMM Research Center for Molecular Medicine of the Austrian Academy of Sciences, 1090 Vienna, Austria; 3 Department of Orthopaedics, Medical University of Vienna, 1090 Vienna, Austria; 4 Department of Laboratory Medicine, Medical University of Vienna, 1090 Vienna, Austria; 5 Center of Pathobiochemistry and Genetics, Institute of Medical Genetics, Medical University of Vienna, 1090 Vienna, Austria; 6 Department of Medicine VI, Wilhelminenspital, 1160 Vienna, Austria; 7 Institute of Musculoskeletal Medicine, University Hospital Muenster, 48149 Muenster, Germany; 8 Center for Physiology and Pharmacology, Medical University of Vienna, 1090 Vienna, Austria; 9 Sigmund Freud Private University, Medical School, 1020 Vienna, Austria

**Keywords:** rheumatoid arthritis, fibroblast-like synoviocytes, tumor necrosis factor, mechanistic target of rapamycin, signal transducer and activator of transcription 1, nuclear factor ‘kappa-light-chain-enhancer’ of activated B cells, amino acids, SLC38A9

## Abstract

Accumulating evidence suggests that metabolic master regulators, including mTOR, regulate adaptive and innate immune responses. Resident mesenchymal tissue components are increasingly recognized as key effector cells in inflammation. Whether mTOR also controls the inflammatory response in fibroblasts is insufficiently studied. Here, we show that TNF signaling co-opts the mTOR pathway to shift synovial fibroblast (FLS) inflammation toward an IFN response. mTOR pathway activation is associated with decreased NF-κB-mediated gene expression (e.g., *PTGS2*, *IL-6*, and *IL-8*) but increased STAT1-dependent gene expression (e.g., *CXCL11* and *TNFSF13B*). We further demonstrate how metabolic inputs, such as amino acids, impinge on TNF-mTORC1 signaling to differentially regulate pro-inflammatory signaling circuits. Our results define a critical role for mTOR in the regulation of the pro-inflammatory response in FLSs and unfold its pathogenic involvement in TNF-driven diseases, such as rheumatoid arthritis (RA).

## Highlights

• mTOR regulates fibroblast-like synoviocyte inflammatory transcriptional programs
• mTOR limits NF-κB signaling by enhancing IκB-α
• mTOR shifts inflammation toward STAT1-dependent genes
• mTOR couples amino acid sensing to TNF activation in fibroblast-like synoviocytes

## Graphical Abstract

**Figure:**
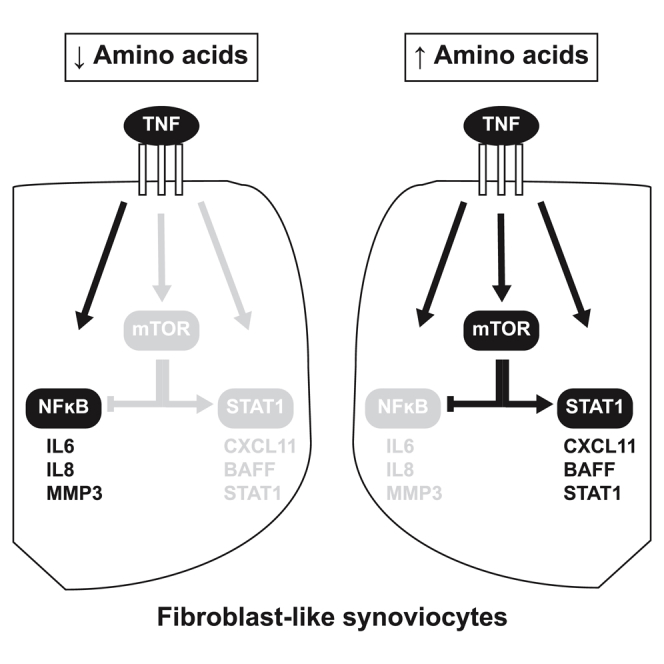

## Teaser

Karonitsch et al. show that TNF signaling co-opts the mTOR pathway in fibroblast-like synoviocytes. mTOR activation is associated with decreased NF-κB-mediated but increased STAT1-dependent gene expression. Thus, the metabolic checkpoint kinase mTOR regulates the synovial tissue response to inflammation in rheumatoid arthritis (RA).

## Introduction

The mechanistic target of rapamycin (mTOR) is engaged in a variety of cellular functions at the interface of cell metabolism, growth, and differentiation (Laplante and Sabatini, 2012). mTOR is the core catalytic component of two distinct functional protein complexes: mTORC1 regulates key cellular processes, including cell growth, protein synthesis, and autophagy, whereas mTORC2 has been implicated in actin-cytoskeletal organization and cell survival. Nutrients, especially amino acids (aas), and growth factors are the best-known factors that regulate mTORC1 activity. mTORC2 is not affected by nutrients but responds to growth factors (Ma and Blenis, 2009, Oh and Jacinto, 2011). Consistent with its role in major cellular processes, mTOR is also recognized as a key regulator in immune cell activation. mTOR senses intra- and extracellular nutrients, growth factors (GM-CSF), cytokines (e.g., interleukin [IL]-4 and IL-15) and pathogen-associated molecular patterns (e.g., Toll-like receptor 4 [TLR4] agonists) to bioenergetically regulate and optimize effector functions (e.g., proliferation and cytokine expression) of immune cells (e.g., macrophages) (Weichhart et al., 2015). Compared to leucocytes, mTOR’s role in regulating the fibroblast response to inflammation is insufficiently explored (Perl, 2016). However, models of arthritis increasingly stress the role of fibroblasts for defining distinct responses resulting in chronic inflammation and tissue destruction (Buckley, 2011). In particular, fibroblast-like synoviocytes (FLSs) have been shown to act as the primary pro-inflammatory effector cells in rheumatoid arthritis (RA). In response to pro-inflammatory mediators, most notably tumor necrosis factor (TNF), FLSs proliferate, which may cause synovial hyperplasia and pannus formation (Bartok and Firestein, 2010, Kiener et al., 2010). TNF also induces the expression of tissue-degrading enzymes (e.g., matrix metalloproteinases [MMPs]), thereby contributing to the destruction of the extracellular synovial matrix and the articular cartilage (Dayer et al., 1985). Furthermore, TNF-stimulated FLSs secrete vast amounts of cytokines and chemokines (Jones et al., 2017, Nguyen et al., 2017). This cytokine-stimulated production of inflammatory mediators provides a means by which FLSs support the activation and differentiation of infiltrating immune cells (Noss and Brenner, 2008). Previous studies showed that mTOR might contribute to erosive arthritis by controlling FLS invasion (Laragione and Gulko, 2010) and proliferation (Saxena et al., 2011). Due to its pleiotropic role in immune cell activation, we hypothesized that mTOR might affect other TNF-mediated pro-inflammatory effector functions (e.g., chemokine or cytokine production).

## Results

### mTOR Activation in the Rheumatoid Synovium

**Figure 1:**
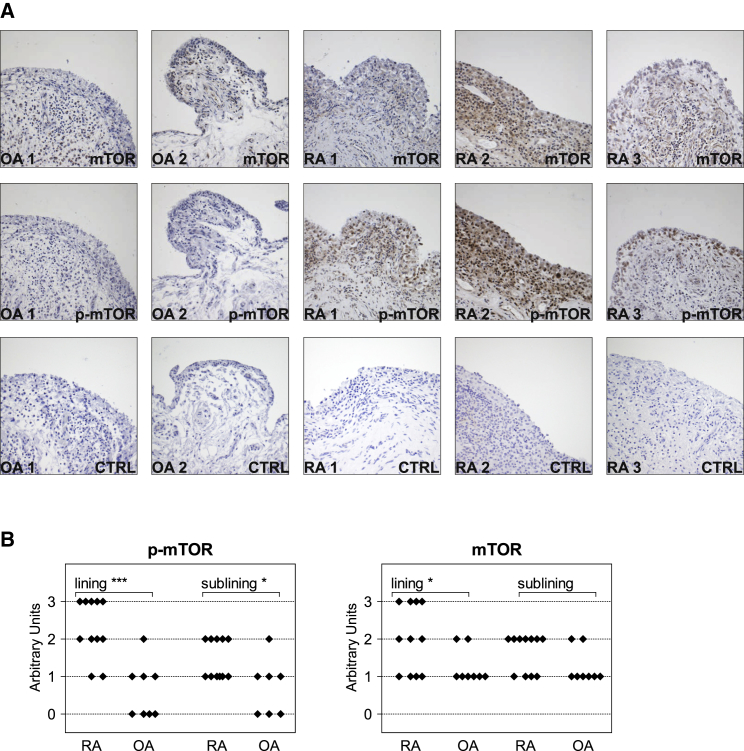
Activation of mTOR in RA (A) Immunohistochemistry showing phosphorylated (p)-mTOR (S2448) and mTOR expression in paraffin-embedded synovial tissue sections from patients suffering from osteoarthritis (OA) or rheumatoid arthritis (RA). Synovial tissue sections were also stained with an isotype-matched control antibody (CTRL). (B) RA (n = 12) and OA (n = 8) synovial tissue sections were evaluated for p-mTOR and mTOR expression using a semiquantitative score (0 = no staining, 3 = high staining). ^∗∗∗^p = 0.0002 and ^∗^p < 0.05, unpaired Student’s t test. See also Table S1.

**Figure 2:**
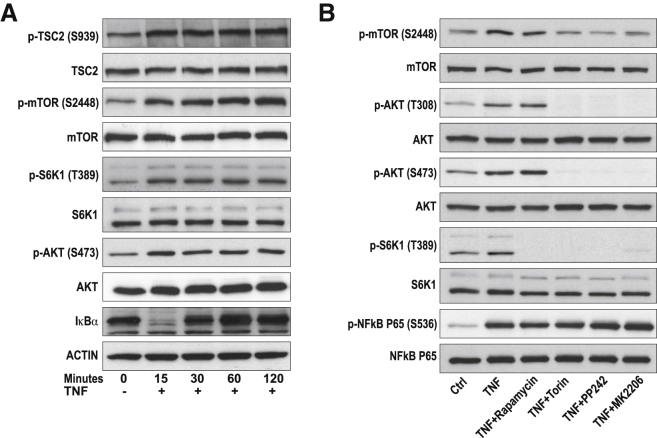
TNF Activates the mTOR Pathway in RA-FLSs (A) Immunoblots of TNF-treated (10 ng/mL) RA fibroblast-like synoviocytes (RA-FLSs). Representative blots for five independent experiments with FLS cell lines from five donors are shown. (p)-S6K: upper band shows P85-S6K, and lower band shows the P70-S6K isoform. (B) RA-FLSs were preincubated with DMSO (Ctrl), rapamycin (250 nM), torin (250 nM), PP242 (1,000 nM), or MK2206 (1,000 nM) for 60 min and then stimulated with TNF (10 ng/mL) for 15 min. Blots are representative for three independent experiments with RA-FLS cell lines from three donors. See also Figure S1.

To examine the degree and pattern of mTOR activation within the inflamed synovium, RA (n = 12) and osteoarthritis (OA, n = 8) synovial tissue samples (clinical and demographic characteristics are presented in Table S1) were stained with antibodies to phosphorylated (p)-mTOR. Immunohistochemistry revealed increased mTOR activity in RA synovial tissues when compared to OA (Figure 1). mTOR activity was preferentially detected in the hyperplastic synovial lining layer and in fibroblast-like cells in the sublining area, pointing toward enhanced mTOR activity in RA-FLSs. No associations between type or dose of immunosuppressant drugs and mTOR or p-mTOR expression in RA synovial tissues were observed. To study whether pro-inflammatory mediators contribute to mTOR activation in RA, cultured RA-FLSs were exposed to TNF. TNF stimulation promoted the inactivation (phosphorylation) of TSC2, a downregulator of mTOR activity (Laplante and Sabatini, 2012). The phosphorylation or activation of mTOR and of both isoforms (P70 and P85) of the mTORC1 substrate S6K1 (Figure 2A) was increased in TNF-treated RA-FLSs compared to unstimulated RA-FLSs. TNF also induced AKT S473 phosphorylation (Figure 2A), which is highly associated with mTORC2 activity (Sarbassov et al., 2005). TNF stimulation did not further increase the phosphorylation of the mTORC1 target 4E-BP1 (Figure S1).

The TNF-induced activation of AKT and S6K1 could be blocked by Torin-1 and PP242 (Figure 2B), two specific inhibitors of both mTORC1 and mTORC2 functions (Liu et al., 2012), indicating that TNF specifically activates AKT and S6K1 via mTOR. Rapamycin, which is selective for mTORC1, specifically prevented the phosphorylation of the mTORC1 substrate S6K1, while the mTORC2-dependent phosphorylation of AKT on S473 was not affected (Figure 2B).

Growth factors are known to activate mTOR via AKT, a kinase stimulated through a dual phosphorylation mechanism: T308 by PDK1 and S473 by mTORC2 (Zoncu et al., 2011). To elucidate the role of AKT in the TNF-mTOR pathway, TNF stimulated RA-FLSs were concomitantly treated with the selective AKT inhibitor MK2206. MK2206 prevented the TNF-induced activation of mTOR and S6K1 (Figure 2B), demonstrating that TNF activates AKT to subsequently control mTOR activity in RA-FLSs.

These data reveal increased activity of mTOR in rheumatoid synovitis and show how the pro-inflammatory microenvironment may affect mTOR activity in RA-FLSs.

### mTOR Modulates the Gene Expression Response to TNF

**Figure 3:**
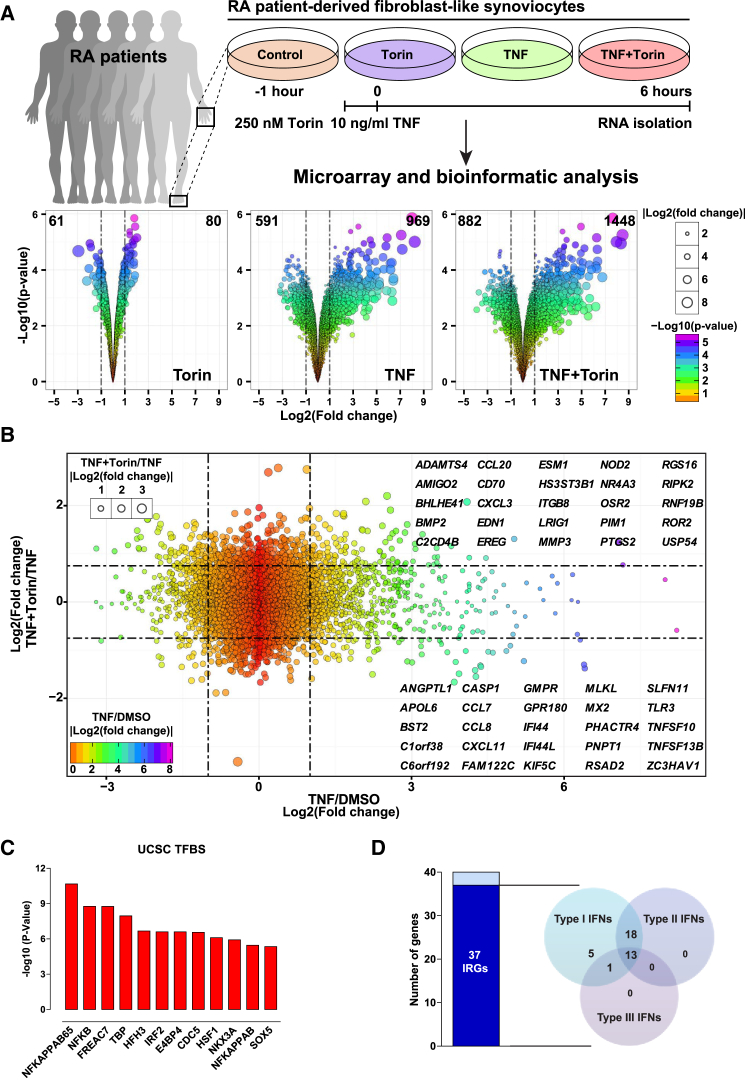
mTOR Modulates the Gene Expression Response to TNF in FLSs (A) Schematic illustration of the microarray experiment. RA-FLSs from five donors were pretreated with either DMSO (Ctrl) or torin (250 nM) for 1 hr and then stimulated with TNF (10 ng/mL) for 6 hr. Volcano plots show the magnitude of differential expressed transcripts for each treatment. Differentially regulated genes were identified by the significance analysis of microarrays (SAM) (fold induction > 2, FDR < 0.01). The number of differentially regulated transcripts in comparison with DMSO (Ctrl) treatment is depicted. (B) Scatterplot showing the influence of mTOR inhibition (by Torin-1) on TNF-upregulated transcripts. The 25 most significant genes are shown in the upper-right and lower-right quadrant. (C) The top 12 transcription factor binding sites (TFBSs) that are enriched in the TNF-upregulated genes that were positively regulated by the pretreatment with Torin-1 (DAVID) (https://david.ncifcrf.gov/home.jsp). (D) mTOR inhibition decreased the TNF-induced expression of 40 genes. This group was significantly enriched for interferon-regulated genes (IRGs) (92.5% of all genes [37/40]) (http://www.interferome.org/interferome/home.jspx). The bar graph shows the number of genes that were classified as IRGs. See also Figures S2 and S4.

To decipher cellular processes that are regulated by mTOR, changes in the global transcriptome in RA-FLSs that were exposed to TNF in the presence or absence of Torin-1 were measured. While Torin-1 alone had minor effects on transcription, TNF increased the expression of 969 transcripts at least 2-fold (Figure 3A). As expected, TNF-stimulated RA-FLSs showed increased expression of genes encoding pro-inflammatory cytokines or chemokines (e.g., *IL-6*, *IL-8*, *CCL20*, *CXCL11*, and *TNFSF13B*), proteolytic enzymes (e.g., *MMP1* and *MMP3*), and other key molecules involved in RA pathogenesis, such as prostaglandin-endoperoxide synthase 2 (*PTGS2*). Inhibition of mTOR by Torin-1 augmented the expression of a subset of TNF-inducible genes (TNF+TOR/TNF, 124 transcripts; cutoff, log2 > 0.75) (Figure 3B), indicating that the TNF-induced expression of these genes (e.g., *CCL20*, *IL-6*, *IL-8*, *MMP1*, *MMP3*, and *PTGS2*) may be negatively affected by mTOR. Gene set enrichment analysis revealed that most of these genes are likely controlled by the transcription factor nuclear factor κB (NF-κB) (Figure 3C), indicating that mTOR inhibits NF-κB activity. However, Torin-1 prevented the TNF-induced expression of 63 transcripts (42 genes). This group was significantly enriched for interferon-regulated genes (IRGs) (Figure 3D), suggesting that mTOR function is required for the TNF-induced expression of IRGs. Many of these genes, including *TNFSF13B* (Ohata et al., 2005) and *CXCL11* (Ueno et al., 2005), are abundantly expressed in the rheumatoid synovium. Differentially expressed candidate genes were validated by qPCR (Figure S2).

These data suggest a hitherto-unknown modulatory role for mTOR in response to TNF: mTOR attenuates the inflammatory response mediated via NF-κB but specifically promotes transcription programs responsible for the TNF-induced expression of IRGs.

### Validation Using a 3D Tissue Culture System

**Figure 4:**
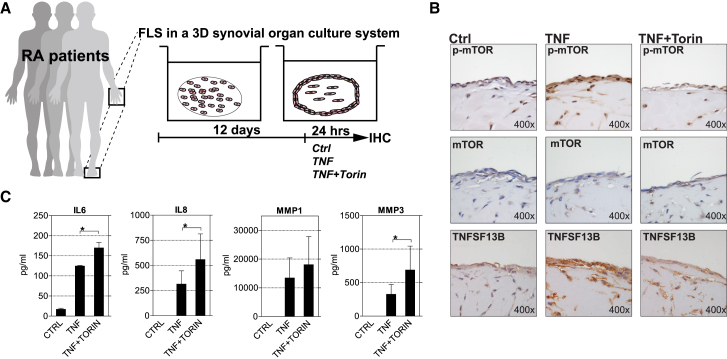
Validation Using a 3D Tissue Culture System (A) Schematic illustration of the 3D tissue culture experiment. RA-FLSs were cultured in micromass organ cultures for 12 days. After serum starvation (overnight), FLSs were treated with DMSO (Ctrl), TNF (10 ng/mL), or TNF (10 ng/mL) + torin (250 nM) for 24 hr. (B) Micromasses were fixed, sectioned, and stained with hematoxylin and specific antibodies for p-mTOR (S2448), mTOR, and TNFSF13B. Representative pictures of three independent experiments performed with RA-FLSs from three donors are shown. For isotype controls, see Figure S3. (C) ELISA of IL-6, IL-8, MMP1 and MMP3 in culture supernatants of micromass organ cultures. Values are the mean ± SEM of three independent experiments that were performed with RA-FLSs from three donors. ^∗^p < 0.05, paired t test.

In contrast to conventional two-dimensional (2D) cultures, three-dimensional (3D) culture systems allow for proper, near-native cellular organization, making cellular processes such as protein expression and cell signaling events more likely to reflect *in vivo* situations. We therefore employed an *ex vivo* 3D tissue culture system to validate the transcriptomic data and to further analyze the role of mTOR in synovial inflammation. This model system was previously shown to faithfully recapitulate many *in vivo* functions of the synovial membrane (Kiener et al., 2010). RA-FLSs were placed into a Matrigel matrix, and the mixture was cultured as a floating sphere. Over time, the RA-FLSs spontaneously organized a synovial-like tissue structure with two separate layers: a lining layer at the interface between the fluid phase (culture medium) and the matrix and a sublining layer with few scattered FLSs within in the matrix (Figures 4A and 4B). TNF-stimulation of the tissue-spheres resulted in the expression of IL-6, IL-8, MMP1, MMP3 (Figure 4C), and TNFSF13B (Figure 4B) (see Figure S3 for isotype controls). The TNF-treated synovial organoid also displayed increased levels of p-mTOR (Figure 4B) (see Figure S3 for isotype controls), similar to RA synovial tissues (Figure 1). The addition of Torin-1 prevented the TNF-induced activation of mTOR (Figure 4B). Consistent with the microarray data, mTOR inhibition promoted the expression of the NF-κB targets IL-6, IL-8, MMP1, and MMP3 (Figure 4C) but decreased the expression of the IRG TNFSF13B (Figure 4B) in TNF-treated RA-FLSs.

To exclude Torin-1 off-target effects, we next determined the impact of PP242 on TNF-induced gene and protein expression (Figures S4A and S4B). Similar to Torin-1, PP242 promoted the TNF-induced expression of NF-κB targets (*PTGS2* and *CCL20*) and decreased the expression of IRGs (*CXCL11* and *TNFSF13B*). To exclude that our experimental observations (e.g., cellular response to TNF or mTOR inhibition) are due to intrinsic defects (e.g., somatic mutations) in RA-FLSs, we next stimulated OA-FLSs with TNF in the presence or absence of mTOR inhibitors. Similar effects of mTOR inhibition were observed for both RA-FLSs and OA-FLSs, suggesting that there is no distinct disease-related response of FLSs (Figures S4C and S4D).

### mTOR Limits NF-κB Signaling by Influencing IκB-α Dynamics

**Figure 5:**
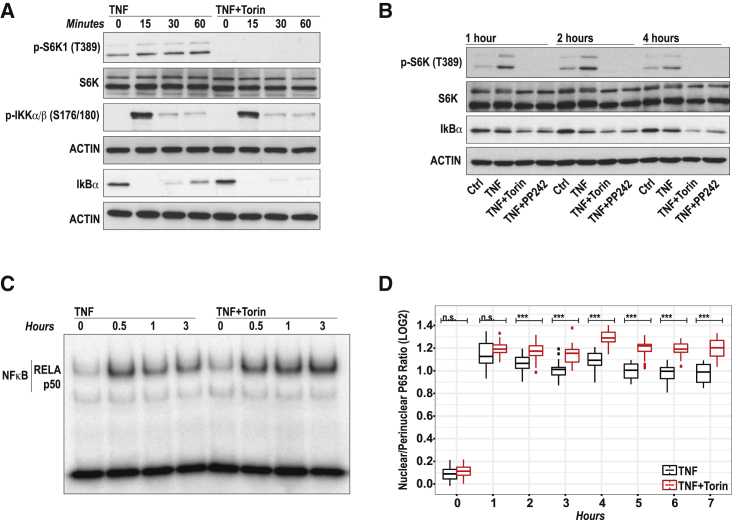
mTOR Affects NF-κB Signaling by Influencing IκB-α Dynamics (A and B) Western blots of RA-FLSs that were pretreated with DMSO (Ctrl, TNF), torin (250 nM), or PP242 (1,000 nM) for 60 min and then stimulated with TNF (10 ng/mL). Representatives blots of five (A) or three (B) independent experiments with RA-FLSs from different donors are shown. For quantification of western blots, see Figure S5. (C) NF-κB DNA binding activity by EMSA in nuclear extracts from RA-FLSs, which were treated with DMSO or Torin-1 (250 nM) 1 hr before TNF stimulation (10 ng/mL). Representative blots of four independent experiments performed with RA-FLS cell lines from four donors are shown. For quantification of EMSA, see Figure S5. (D) Boxplots displaying the log2 nuclear-to-perinuclear P65 signal ratio calculated from automatically captured and analyzed images of RA-FLSs. FLSs were treated with either DMSO or 250 nM torin for 60 min before TNF stimulation (10 ng/mL). Pooled data from 6 RA-FLS cell lines are shown. Unpaired Student’s t test has been used to assess the statistical significance of treatment differences. See also Figure S6.

NF-κB is a pivotal transcription factor in synovial inflammation (Blüml et al., 2014). Therefore, it is intriguing that mTOR acts as a negative regulator of NF-κB-regulated gene expression in FLSs. To mechanistically explore the Torin-1 effect, we asked whether mTOR affects the TNF-induced activation of the NF-κB signaling pathway. However, well-known readouts for canonical NF-κB signaling, such as the phosphorylation of IKKα/β (Figure 5A), P65 or P105 (Figure S5A) were not impaired by Torin-1. Instead, both Torin-1 and PP242 (Figures 5A and 5B; Figures S5B and S5C) markedly suppressed the re-appearance of the NF-κB inhibitor IκB-α. Newly synthesized IκB-α is known to enter the nucleus to remove NF-κB from the DNA and bring the NF-κB complex back to the cytoplasm (Ghosh and Hayden, 2012). Lower IκB-α protein levels in Torin-1-treated FLSs would therefore suggest nuclear accumulation of NF-κB. Both electrophoretic mobility shift assay (EMSA) (Figure 5C; Figure S5D) and immunofluorescence confocal microscopy of P65 nuclear translocation (Figure 5D; Figure S6) revealed increased nuclear abundance of NF-κB in TNF-treated RA-FLSs that were concomitantly treated with Torin-1. Altogether, these results demonstrate that mTOR controls the expression of NF-κB-regulated genes by affecting IκB-α-mediated P65 translocation.

### mTOR Regulates IRG Expression by Promoting the Expression and Activation of STAT1

**Figure 6:**
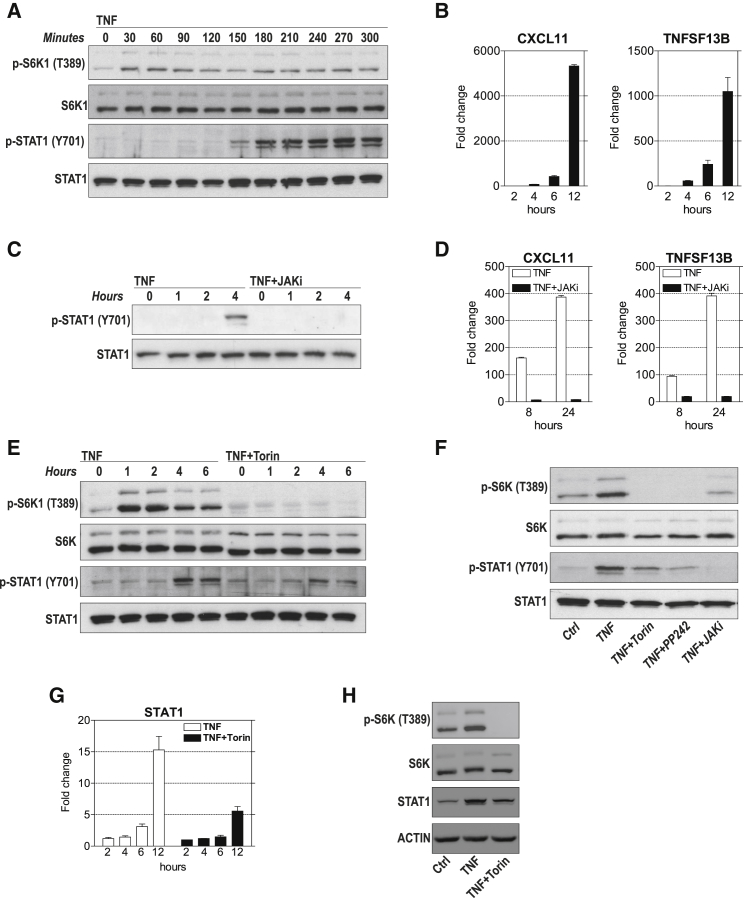
mTOR Promotes TNF-Induced Expression and Activation of STAT1 (A) Immunoblots of S6K1, p-S6K1, Stat1, and p-Stat1 in human RA-FLSs treated with TNF (10 ng/mL). Representative blots of four independent experiments performed with FLSs from four donors are shown. STAT1: upper band shows STAT1α (91 kDa), and lower band shows STAT1β (84 kDa). (B) RA-FLSs were stimulated with TNF (10 ng/mL). qPCR was performed in technical triplicates (error bars, SEM of triplicates). Representative graphs of five independent experiments with different RA-FLS cell lines are shown. mRNA expression is presented relative to untreated cells. (C and D) RA-FLSs were pretreated with JAK inhibitor I (300 nM) for 1 hr and then stimulated with TNF (10 ng/mL). (C) Immunoblots of RA-FLSs. Representative blots of three independent experiments with different RA-FLS cell lines are shown. (D) Gene expression was determined by qPCR. qPCR was performed in technical triplicates (error bars, SEM of triplicates). Representative graphs of two independent experiments with different RA-FLS cell lines are shown. mRNA expression is presented relative to that in untreated cells. (E) Immunoblots of FLSs stimulated with TNF (10 ng/mL) that were pretreated with Torin-1 (250 nM) or DMSO. Representative blots of three independent experiments are shown. (F) Immunoblots of RA-FLSs that were pretreated with DMSO, torin (250 nM), PP242 (1,000 nM), or Jak inhibitor I (300 nM) for 1 hr and then stimulated with TNF (10 ng/mL) for 3 hr. Representative blots of three independent experiments are shown. (G) *STAT1* expression in RA-FLSs as determined by qPCR. RA-FLSs from three donors were pretreated with Torin-1 (250 nM) or DMSO for 1 hr and then stimulated with TNF (10 ng/mL). Bars show mean ± SEM. Expression is presented relative to that in DMSO-treated cells. (H) Immunoblots of RA-FLSs pretreated with DMSO or Torin-1 (250 nM) for 1 hr and then stimulated with TNF (10 ng/mL) for 24 hr. Representative blots of at least six independent experiments are shown.

The signaling circuit that allows TNF to induce the expression of IRGs in macrophages and endothelial cells depends on an interferon (IFN)β-mediated autocrine loop that activates the transcription factor STAT1 to induce the expression of IRGs (Venkatesh et al., 2013, Yarilina et al., 2008). To address how mTOR interferes with the expression of IRGs, we first examined whether this signaling cascade exists in RA-FLSs. TNF stimulation of RA-FLSs resulted in the activation of STAT1 within 3 hr (Figure 6A). Correspondingly, time course experiments revealed a delayed generation of TNF-induced IRGs (Figure 6B). Inhibition of Janus kinases (JAKs), which signal upstream of STATs (Schwartz et al., 2016), prevented the TNF-induced activation of STAT1 (Figure 6C) and decreased the expression of IRGs (Figure 6D). Knowing that the TNF-induced expression of IRGs depended on STAT1 activation, we determined whether mTOR interferes with STAT1 phosphorylation. Both Torin-1 and PP242 decreased the TNF-induced phosphorylation of STAT1 (Figures 6E and 6F). TNF is also known to increase total STAT1 protein levels in FLSs (Sohn et al., 2015). Higher levels of total STAT1 are associated with enhanced signal transduction and expression of IRGs (Lehtonen et al., 1997). We therefore determined whether mTOR affects TNF-induced total STAT1 expression. Torin-1 inhibited the TNF-induced expression of STAT1 in RA-FLSs (Figures 6G and 6H). Altogether, these results define a critical role for mTOR in the TNF-induced activation and expression of STAT1, which is critical for TNF-induced IRG expression in FLSs.

### Control of TNF-Induced Gene Expression by Amino Acids

**Figure 7:**
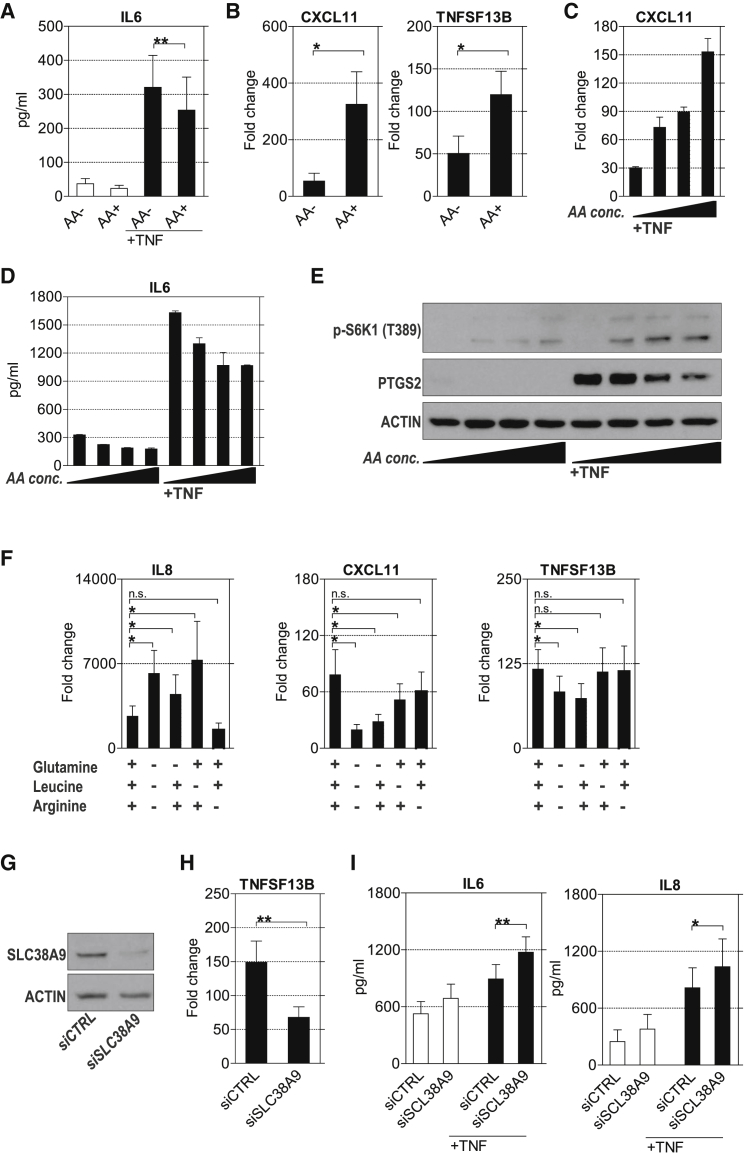
Control of TNF-Induced Gene Expression by Amino Acids (A and B) RA-FLSs cultured in DMEM with or without amino acids (aas) were stimulated with TNF (10 ng/mL) for 6 hr. (A) Levels of IL-6 in the supernatants were measured by ELISA. Values are the mean ± SEM. ^∗∗^p < 0.01, Student’s paired t test, n = 5. (B) Expression of *CXCL11* and *TNFSF13B* was determined by qPCR. Expression is presented relative to that in untreated cells. Values are the mean ± SEM. ^∗^p < 0.05, Student’s paired t test, n = 4. (C–E) RA-FLSs were cultured in aa-free DMEM or DMEM that was reconstituted with amino acids (1/3× aa, 2/3× aa, 3/3× amino acid of standard amino acid concentration) and then stimulated with 10 ng/mL of TNF for 6 hr. (C) *CXCL11* gene expression was determined by qPCR. Values are the mean ± SD of technical replicates. A representative of three independent experiments is shown. Expression is presented relative to unstimulated cells. (D) IL-6 concentration in supernatants was determined with ELISA. Values represent the mean ± SD of technical replicates. A representative of three independent experiments is shown. (E) Immunoblots of whole-cell lysates using anti-PTGS2 and anti-p-S6K antibodies. Representative blots of three independent experiments are shown. (F) RA-FLSs (n = 7) cultured in DMEM containing or lacking glutamine, leucine, and/or arginine were stimulated with TNF for 6 hr. Gene expression was determined by qPCR. Expression is presented relative to that in untreated cells. Values are the mean ± SEM. ^∗^p < 0.05, Student’s paired t test. (G) Immunoblots of RA-FLSs after transfection with SLC38A9 or non-targeting control siRNA. (H and I) Transfected FLSs from five donors suffering from RA were treated with TNF (10 ng/mL) for 6 hr (H). *TNFSF13B* gene expression was determined by qPCR. Expression is presented relative to that in unstimulated cells. (I) Transfected RA-FLSs (n = 5) were treated with TNF (10 ng/mL) for 6 hr. Supernatants were analyzed by ELISA. Values are the mean ± SEM. ^∗^p < 0.05 and ^∗∗^p < 0.01, Student’s paired t test.

The amino acids are the most potent regulators of mTORC1 activity, whereas mTORC2 is not sensitive to nutrients (Laplante and Sabatini, 2012). We therefore varied total amino acid concentrations as another way to modulate mTOR activity and to dissect the relative contributions of mTORC1 versus mTORC2 in regulating TNF-induced gene expression. Deprivation of amino acids increased the TNF-induced expression of IL-6 (Figures 7A and 7D), MMP3 (data not shown), and PTGS2 (Figure 7E), while the expression of IRGs (e.g., *CXCL11* and *TNFSF13B*) decreased (Figures 7B and 7C). Titrating amino acids also affected the TNF-induced activation of mTORC1, as indicated by the phosphorylation of S6K1 (Figure 7E). The main amino acids that activate mTOR are glutamine, leucine, and arginine (Durán et al., 2012, Jewell et al., 2013, Nicklin et al., 2009, Rebsamen and Superti-Furga, 2016). Culture in DMEM lacking glutamine, leucine, and arginine increased the TNF-induced expression of *IL-8*, while the expression of IRGs (*CXCL11* and *TNFSF13B*) was decreased (Figure 7F). FLSs were also cultured in medium lacking just one of these three amino acids. Depletion of glutamine increased the expression of IL-8 but decreased the TNF-induced expression of *CXCL11* and *TNFSF13B* (Figure 7F). Arginine deficiency did not affect TNF-induced gene expression, while leucine depletion resulted in increased *IL-8* but decreased *CXCL11* expression (Figure 7F). To confirm that mTORC1 integrates metabolic and inflammatory cues to control RA-FLS effector functions, we next silenced SLC38A9. SLC38A9 was identified as a component of the lysosomal aa-sensing machinery that couples mTORC1 activation (Rebsamen et al., 2015, Wang et al., 2015) to the abundance of amino acids. Knockdown of SLC38A9 (Figure 7G) resulted in decreased expression of *TNFSF13B* and *CXCL11* (Figure 7H) (data not shown), while the expression of the NF-κB targets, such as IL-6 and IL-8 (Figure 7I), increased. These results indicate that TNF-induced inflammatory programs are differentially regulated by mTORC1 depending on the availability amino acids.

## Discussion

Metabolic key regulators, such as mTOR, are integral to immune cell activation to fuel the highly demanding metabolic needs that are required for effector functions, such as differentiation, proliferation, or cytokine production (Powell et al., 2012, Weichhart et al., 2015). In this respect, mesenchymal stromal cells received less attention. However, their critical role in non-resolving inflammation is emerging (Armaka et al., 2008, Buckley, 2011). FLSs have been recognized as key drivers of synovial inflammation in RA; they secrete vast amounts of cytokines and chemokines that promote immune cell recruitment and activation (Noss and Brenner, 2008). Given the key role of mTOR in immune cell activation and the relative lack of understanding of its function in mesenchymal stromal cells, we aimed to unfold its involvement in FLS activation.

Clinical trials have proved the central role of TNF in the pathogenesis of RA (Smolen and Emery, 2011), and preclinical studies have extended our understanding of how TNF drives the classic pro-inflammatory response of FLSs by activating the NF-κB and mitogen-activated protein kinase (MAPK) pathway (Bartok and Firestein, 2010, Lee et al., 2013a). Based on studies that showed mTOR activation in response to various cytokines (Dan and Baldwin, 2008, Lee et al., 2007a) and encouraged by our immunohistochemical studies, we hypothesized that TNF activates the mTOR pathway in FLSs. TNF robustly increased mTOR activity in FLSs that were grown in conventional 2D cultures or in the 3D synovial organ culture system. While molecular circuits mediating nutrient- and growth factor-induced activation of mTOR have extensively been studied (Laplante and Sabatini, 2012, Rebsamen and Superti-Furga, 2016), few studies have explored the mechanistic basis of TNF-induced mTOR activation. Depending on the cell type, these studies identified (Dan and Baldwin, 2008, Lee et al., 2007a) AKT-dependent and AKT-independent pathways. For FLSs, we clearly observed an AKT-dependent activation of mTOR upon TNF stimulation.

To explore the consequences of mTOR pathway activation in response to TNF, we employed global gene expression profiling. We found that mTOR activation suppressed NF-κB transcriptional programs. This is in line with previous studies that suggested distinct crosstalks between the mTOR and the NF-κB signaling pathways. Activation of mTOR by loss of the inhibitory TSC1/TSC2 function resulted in decreased NF-κB activity in mouse embryonic fibroblasts (Ghosh et al., 2006). Mechanistically, it was proposed that mTOR interferes with the phosphorylation of principal NF-κB pathway components. For FLSs, we could not demonstrate such effects. Instead, our data suggest that mTOR regulates NF-κB activity by controlling the re-appearance of the NF-κB inhibitor IκB-α. Because inhibition of mTOR promoted the expression of numerous pro-inflammatory and tissue-degrading genes (e.g., *PTGS2*, *IL-8*, or *MMP3*), mTOR-mediated negative regulation of NF-κB signaling might also raise a cautionary note. RA patients treated with the mTOR inhibitor everolimus exhibited an increase in levels of inflammatory markers, such as the erythrocyte sedimentation rate (ESR) (Bruyn et al., 2008). However, arthritis in everolimus-treated patients was ameliorated. Such clinical benefits might be explained by the effects of mTOR inhibitors on FLS proliferation (Saxena et al., 2011) and invasion (Laragione and Gulko, 2010). Our experiments also revealed mTOR-dependent activation of STAT1 in RA-FLSs. Studies suggest that STAT1 pathway activation correlates with severity of inflammation in RA (Ruschpler et al., 2003, Walker et al., 2006). Thus, the therapeutic effect of everolimus may result from decreased STAT1 activity that is mediated by inhibition of mTOR. Numerous STAT1-regulated genes (Rauch et al., 2013), such as *CXCL9*, *CXCL10*, and *CXCL11*, which are crucial for leucocyte homing to inflamed tissues, are abundantly expressed in RA (Lee et al., 2013b). The efficacy of JAK inhibitors that interfere with STAT1 activation further supports the potential involvement of STAT1 and IRGs in RA (Boyle et al., 2015, Schwartz et al., 2016). We and others have identified TNF as the potential driver for the IRGs in RA (Karonitsch et al., 2012, Yarilina et al., 2008). With mTOR, we here introduce a determining factor that regulates the TNF-induced activation of the transcription factor STAT1, which is responsible for the subsequent expression of pro-inflammatory mediators, such as CXCL11 or TNFSF13B.

Why does TNF signaling co-opt the AKT-mTOR pathway to couple metabolic inputs, such as amino acids, to the TNF-induced expression of STAT1-regulated genes? TNFSF13B, also known as B cell-activating factor (BAFF), is a key factor for B cell activation and maturation. TNFSF13B overexpression is associated with autoantibody production (Mackay et al., 1999). TNFSF13B is abundantly expressed in rheumatoid synovial fibroblasts (Nakajima et al., 2007, Ohata et al., 2005) (Bombardieri et al., 2011). In addition, CXCL11 that is chemotactic for activated T cells was reported to be highly expressed in RA (Ueno et al., 2005). Given that amino acids, such as glutamine, are key nutrients for lymphocyte proliferation (Caro-Maldonado et al., 2012, Cobbold et al., 2009, Kouidhi et al., 2017), we propose that mTORC1 controls the TNF-induced expression of STAT1-regulated genes to adapt energetically costly immune responses. Thus, RA-FLSs may act as tissue-resident metabolic sentinel cells that solely promote lymphocyte migration to the inflamed synovium under metabolically favorable conditions. A similar role for mTOR has been suggested for endothelial cells. mTOR inhibition prevented the ability of TNF-activated endothelial cells to capture T cells under conditions of venular flow *in vitro* and reduced leukocyte migration to sites of inflammation *in vivo* (Wang et al., 2014).

We here describe a so far unknown, temporally defined signaling circuit in which TNF activates mTOR to specifically tailor the FLS response to inflammation. Consistent with its role as a key metabolic sensor, mTORC1 couples nutrient availability to RA-FLS effector functions. The identification of mTOR as a critical regulator of the response to TNF in RA-FLSs provides avenues for uncovering the molecular mechanism underlying TNF-driven inflammatory diseases.

## Experimental Procedures

### Isolation and Culture of FLSs

With approval of the local ethics committee, synovial tissues from patients fulfilling the American College of Rheumatology/European League Against Rheumatism (ACR/EULAR) classification criteria for RA (Aletaha et al., 2010) and patients suffering from OA were obtained as discarded specimens following synovectomy or joint replacement. Culture of FLSs was performed in DMEM (Gibco) supplemented with 10% heat-inactivated fetal bovine serum (FBS) (HyClone) and with 1% penicillin and streptomycin (P/S) and nonessential amino acids (both Gibco) as previously described (Kiener et al., 2009). FLSs between passages 4 and 8 were used for all experiments. The following cytokines, inhibitors, and blocking antibody were used: rhTNF (R&D), JAK inhibitor I (Calbiochem), rapamycin (Calbiochem), MK2206 (Selleckchem), PP242 (Sigma), and Torin-1 (Tocris). All experiments were repeated with FLS cell lines from different donors.

To study the effects of amino acid deprivation, aa-free DMEM (Pan Biotech) was reconstituted with amino acids (Sigma) except the amino acid or amino acids to be omitted. The culture medium (DMEM ± aa) was changed 1 hr before TNF stimulation.

### Immunohistochemistry

For immunohistochemistry, synovial tissues, RA (n = 12), and OA (n = 8) (patient characteristics are shown in Table S1) or synovial micromass cultures were fixed with paraformaldehyde and embedded in paraffin. Paraffin sections were treated with Tris-EDTA (pH 9). After blocking with goat serum, sections were incubated with primary antibodies (anti-BAFF [TNFSF13B, Enzo Life Sciences] and mTOR or p-MTOR [S2448, both Cell Signaling Technology]) or non-immune immunoglobulins of the same isotype and concentration as the primary antibody (isotype control, anti-rat immunoglobulin [Ig] M [Invitrogen/Thermo Fisher Scientific], and anti-rabbit IgG [R&D]). After incubation with a biotinylated goat anti-rabbit antibody (Vector), sections were incubated with Vectastain Elite reagent and visualized using 3,3-diaminobenzidine (Vector). Sections were counterstained with hematoxylin (Merck). Pictures were taken with an Axioskop 2 microscope (Zeiss) equipped with a digital camera (Olympus).

### Western Blot

FLSs were lysed in radioimmunoprecipitation (RIPA) buffer supplemented with phosphatase and protease inhibitors (Roche). Protein extracts were separated by electrophoresis, followed by electrotransfer onto nitrocellulose membrane. After blocking, membranes were incubated with primary antibodies and then exposed to horseradish peroxidase (HRP)-conjugated secondary antibodies (Jackson Laboratory). Specific bands were detected with the enhanced chemiluminescence (ECL) detection kit (Pierce) on Amersham Hyperfilm ECL (GE Healthcare). Reblots were performed using ReBlot Plus Strong Antibody Solution (Millipore). Primary antibodies used were against actin (Cytoskeleton); AKT, p-AKT (S473), p-AKT (T308), p-IKKα/β (S176/180), p-IκB-α (S32/36), IRF1, mTOR, p-mTOR (S2448), p-NF-κB P65 (S536), p-NF-κB P105 (S933), NF-κB P105, p-S6K1 (T389), PTGS2, STAT1, p-STAT1 (Y701), and p-TSC2 (S939) (Cell Signaling Technology); IκB-α, NF-κB P65, and S6K1 (Santa Cruz); SLC38A9 (Sigma); and tubulin (Abcam). FLS lysis and western blotting procedures for SLC38A9 are described elsewhere (Rebsamen et al., 2015).

### qPCR

RNA was isolated and reverse transcribed using the RNeasy Mini Kit and Omniscript RT kit (QIAGEN). RNA concentration was measured with a NanoDrop spectrophotometer. qPCR was carried out on a Roche Light Cycler using the SensiMix SYBR kit (Bioline). Results were quantified using the 2 − ΔΔC(t) method and using GAPDH expression levels for normalization. Primer sequences were as follows: CCL20 forward: 5′-CTGGCTGCTTTGATGTCAGT-3′, reverse: 5′-CGTGTGAAGCCCACAATAAA-3′; CXCL11 forward: 5′-GAAGGATGAAAGGTGGGTGA-3′, reverse: 5′-AAGCACTTTGTAAACTCCGATG-3′; GAPDH forward: 5′-TGATGACATCAAGAAGGTGGTGAAG-3′, reverse 5′-TCCTTGGAGGCCATGTGGGCCAT-3′; IL-6 forward: 5′-GTGTGAAAGCAGCAAAGAGG-3′, reverse: 5′-GGCAAGTCTCCTCATTGAATCC-3′; MMP1 forward: 5′-TGTGGACCATGCCATTGAGAA-3′, reverse 5′-TCTGCTTGACCCTCAGAGACC-3′; PTGS2 forward: 5′-CCGGGTACAATCGCACTTAT-3′; reverse: 5′-GGCGCTCAGCCATACAG-3′; STAT1 forward: 5′-GCCAAAGGAAGCACCAGAGCCAAT-3′, reverse: 5′-AGGAGACATGGGGAGCAGGTTGT-3′; and TNFSF13B: forward: 5′-GGAGAAGGCAACTCCAGTCAGAAC-3′; reverse: 5′-CAATTCATCCCCAAAGACATGGAC-3′.

### ELISA

IL-6 and IL-8 ELISA kits were purchased from eBioscience, and MMP1 and MMP3 ELISA kits were purchased from R&D. Assays were performed according to the recommendations of the manufacturer.

### EMSA

Nuclear extracts from FLSs were prepared and EMSA was performed as described elsewhere (Herdy et al., 2012).

### siRNA-Mediated Knockdown

Lipofectamine RNAiMAX (Invitrogen) and 50 nM small interfering RNA (siRNA) pools (Thermo Fisher Scientific) were used for siRNA-mediated knockdown of SLC38A9 (Rebsamen et al., 2015, Rosner et al., 2010).

### Synovial Micromass Cultures

The 3D *in vitro* culture model of the synovial tissue was performed as previously described (Kiener et al., 2009, Lee et al., 2007b). FLSs were suspended ice-cold in Matrigel Matrix (BD Biosciences). The cell or electron cryomicroscopy (ECM) suspension was placed on polyhydroxyethylmethacrylate (poly-HEMA) (Sigma)-coated culture dishes and overlaid with culture medium: DMEM supplemented with 5% FBS, 1% insulin-transferrin-selenium (ITS) liquid media supplement (Sigma), 0.125% BSA (Calbiochem), 0.008 g ascorbic acid (Fluka), 1% non-essential amino acids (NEAAs) (Gibco), and 1% P/S. Medium was changed twice weekly. RA-FLSs were cultured in the micromass organ cultures for 12 days. After serum starvation overnight, micromasses were treated as indicated. Then, micromasses were fixed, sectioned, and stained as described earlier. Supernatants were analyzed with ELISA according to the manufacturer’s protocol.

### Microarray

RNA was isolated with the RNeasy purification kit from QIAGEN. RNA concentration was measured with a NanoDrop spectrophotometer. The quality of total RNA was determined by the 28S/18S rRNA ratio. The GeneChip PrimeView Human Gene Expression Array (Affymetrix) was used according to the manufacturer’s protocols. Fold changes were computed by performing a significance analysis of microarrays (SAM) using the Easy Microarray Analysis package available in the R statistical environment. The analysis parameters for SAM included a paired analysis with a modified t statistic and a false discovery rate (FDR) threshold of 0.01. Downstream analyses were carried out using Bioconductor packages in the R statistical environment. The Database for Annotation, Visualization and Integrated Discovery (DAVID) (https://david.ncifcrf.gov/home.jsp) (da Huang et al., 2009) was used for the enrichment analysis of transcription factor binding sites. IRGs were identified with Interferome v.2.0 (Rusinova et al., 2013).

### Immunofluorescence Staining and Automated Imaging

For high-content imaging, RA-FLSs were cultured on CellCarrier-384 Ultra Microplates (PerkinElmer). After stimulation, FLSs were fixed in 4% formaldehyde and permeabilized with 0.1% Triton X-100. After blocking with 1% BSA, FLSs were stained with primary antibody (anti-NF-κB P65, Abcam) overnight at 4°C. After incubation with a fluorophore-coupled secondary antibody (Cy5 AffiniPure donkey anti-rabbit IgG, Jackson ImmunoResearch) FLSs were counterstained with DAPI and imaged in an Opera Phenix high-content screening system (PerkinElmer). Images from automated imaging were analyzed in CellProfiler v.3.0.0 (http://cellprofiler.org/citations/). Nuclei were detected from the DAPI signal and used to define a nuclear and a perinuclear region. The P65 signal was quantified in both regions, and log2 ratios of nuclear-to-perinuclear mean intensities were calculated for every cell. Plotting and statistical analysis were performed in R v.3.4.3.

### Statistical Analysis

We used unpaired Student’s t test and paired t tests for comparing groups and paired samples, provided that the data followed Gaussian distribution. Paired and unpaired t tests were performed with GraphPad Prism software, unless otherwise indicated.

## Data and Software Availability

The accession number for the microarray expression data reported in this paper is EBI-ArrayExpress: E-MTAB-6457.

## Supplemental Information

Document S1. Figures S1–S6 and Table S1

Document S2. Article plus Supplemental Information
