# Supplementary material for: mTOR Senses Environmental Cues to Shape the Fibroblast-like Synoviocyte Response to Inflammation
Source: Cell Rep. 2018 May 15;23(7):2157–67. doi: 10.1016/j.celrep.2018.04.044 (PMC5972226; doi:10.1016/j.celrep.2018.04.044)
Supplement: Document S1. Figures S1–S6 and Table S1 [file mmc1.pdf]

Cell Reports, Volume *23*

Supplemental Information

mTOR Senses Environmental Cues to Shape the Fibroblast-like Synoviocyte Response to Inflammation

Thomas Karonitsch, Richard K. Kandasamy, Felix Kartnig, Barbara Herdy, Karolina Dalwigk, Birgit Niederreiter, Johannes Holinka, Florian Sevelda, Reinhard Windhager, Martin Bilban, Thomas Weichhart, Marcus Säemann, Thomas Pap, Günter Steiner, Josef S. Smolen, Hans P. Kiener, and Giulio Superti-Furga

Table S1.

	Rheumatoid Arthritis	Osteoarthritis
**Number of patients (n)**	12	8
**Age, mean ± SD (range) years**	53.25±12.97 (35-69)	73.38±18.24 (40-102)
**Female sex, n**	11	5
**CDAI, mean ± SD (range)**	14.03±9.89 (2.3-36.3)	-
**Prednisolone treatment, n**	11	-
**Methotrexate treatment, n**	7	-
**Leflunomide treatment, n**	2	-
**Azathioprin treatment, n**	2	-
**TNF blocker treatment, n**	2	-

Table S1. Related to Figure 1.
Demographic and clinical characteristics of RA and OA patients for immunohistochemical analyses.

Figure S1

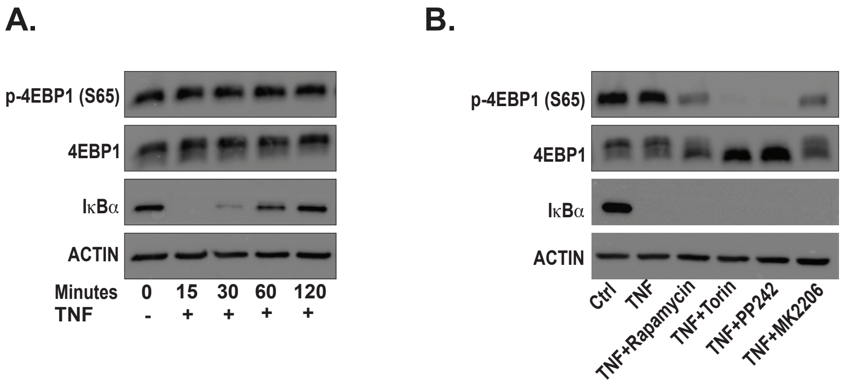

Figure S1. TNF activates the mTOR-pathway in RA-FLS. Related to Figure 2.
A. Immunoblots of TNF treated (10 ng/ml) RA-FLS. Representative blots for three independent experiments with FLS cell lines from three different donors. B. RA-FLS were pre-incubated with DMSO (Ctrl), Rapamycin (250 nM), Torin (250 nM), PP242 (1000 nM) or MK2206 (1000 nM) for 60 minutes and then stimulated with TNF (10 ng/ml) for 15 minutes.
Blots are representative for three independent experiments.

Figure S2

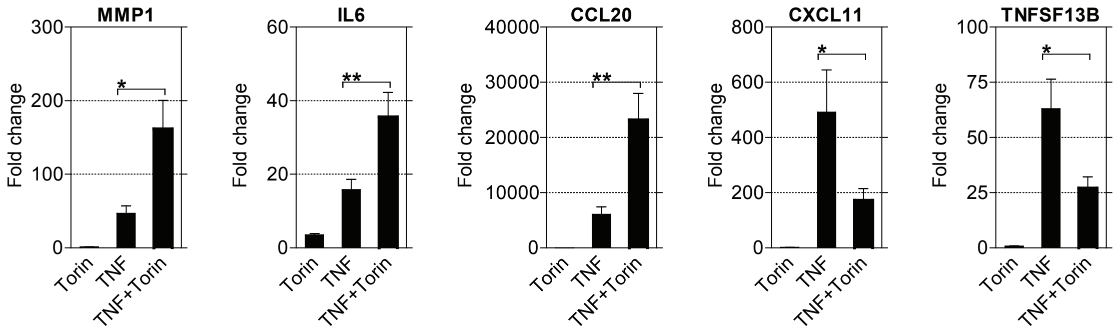

Figure S2. mTOR modulates the gene expression repsonse to TNF in FLS. Related to Figure 3. Validation of microarray data by qPCR. Expression is presented relative to that in DMSO-treated cells. Values are the mean±SEM. *p<0.05, **p<0.01 Student´s paired t-test, n=5.

Figure S3

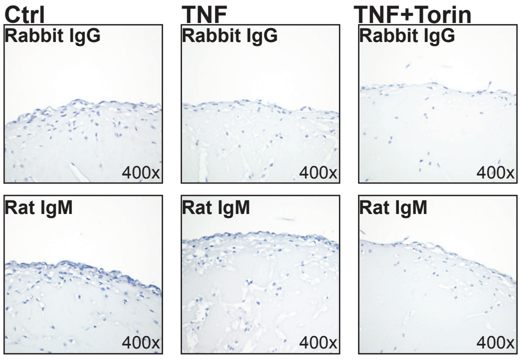

Figure S3. Validation by using a 3-D tissue culture system. Related to Figure 4
Immunohistochemistry images of micromass sections that were stained with isotype control antibodies.

Figure S4

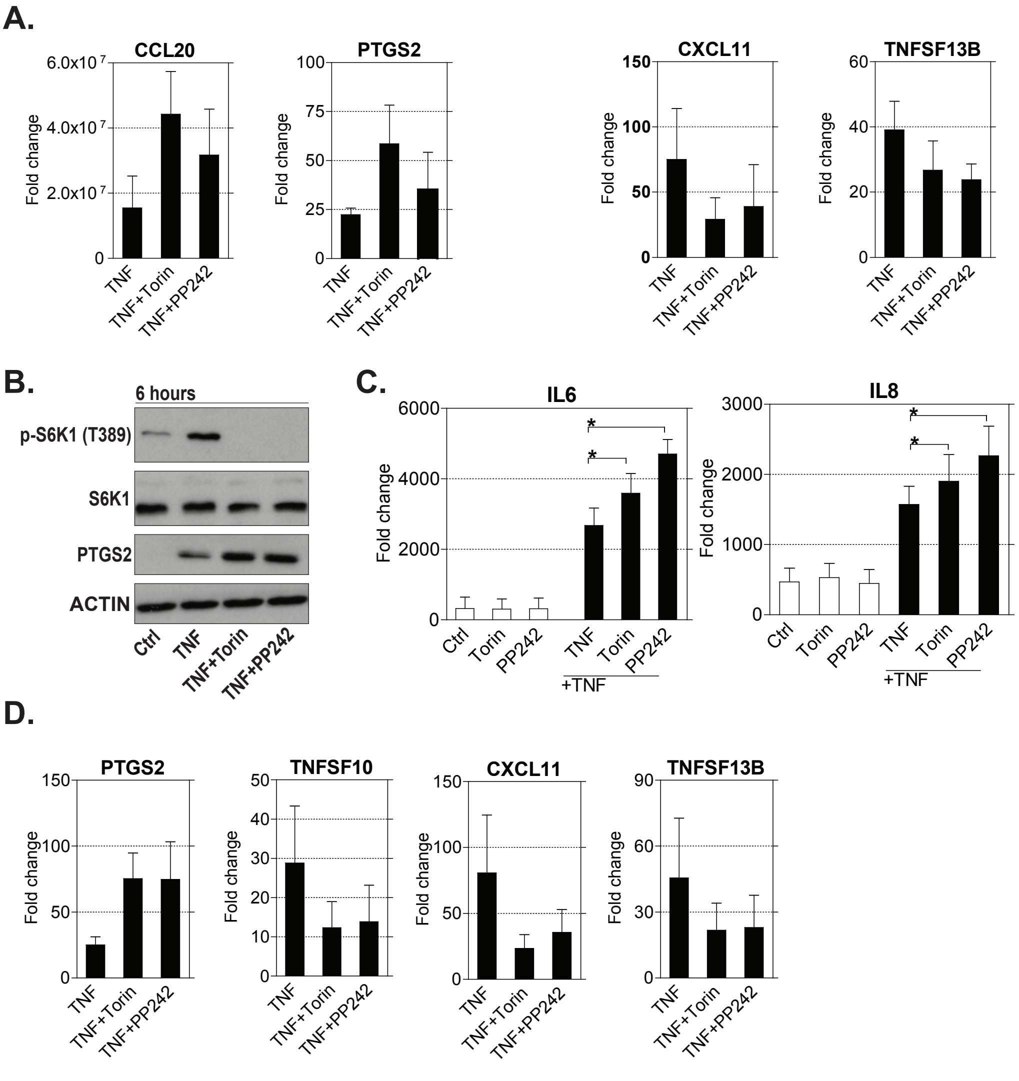

Figure S4. mTOR modulates the gene expression response to TNF in FLS. Related to Figure 3.
A. RA-FLS were pre-treated with either DMSO, Torin (250 nM) or PP242 (1000 nM) for 60 minutes. FLS were then stimulated with TNF (10 ng/ml) for 6 hours. Gene expression was determined by qPCR. Data are from one representative of at least three independent experiments, each performed in technical replicates. Expression is presented relative to that in DMSO treated cells. Values are the mean±SD of technical replicates. B. Western blots of RA-FLS that were pretreated with either DMSO (Ctrl, TNF) Torin (250 nM) or PP242 (1000 nM) for 60 minutes and then stimulated with TNF (10ng/ml) for six hours. Representative blots of three independent experiments with RA-FLS from different donors are shown. C and D. Osteoarthritis (OA) FLS were pre-treated with either DMSO, Torin (250 nM) or PP242 (1000 nM) for 60 minutes. Then OA-FLS were stimulated with TNF (10 ng/ml) for 6 hours. C. Concentration of IL-6 and IL-8 in cell culture supernatants was determined by ELISA. Values are the mean±SEM (n=4), *$p<0.05$, Student´s paired t-test. D. Expression of mRNA for PTGS2, CXCL11 and TNFSF13B was determined by qPCR. mRNA expression is presented relative to that in DMSO treated cells. Values are the mean±SEM (n=4).

Figure S5

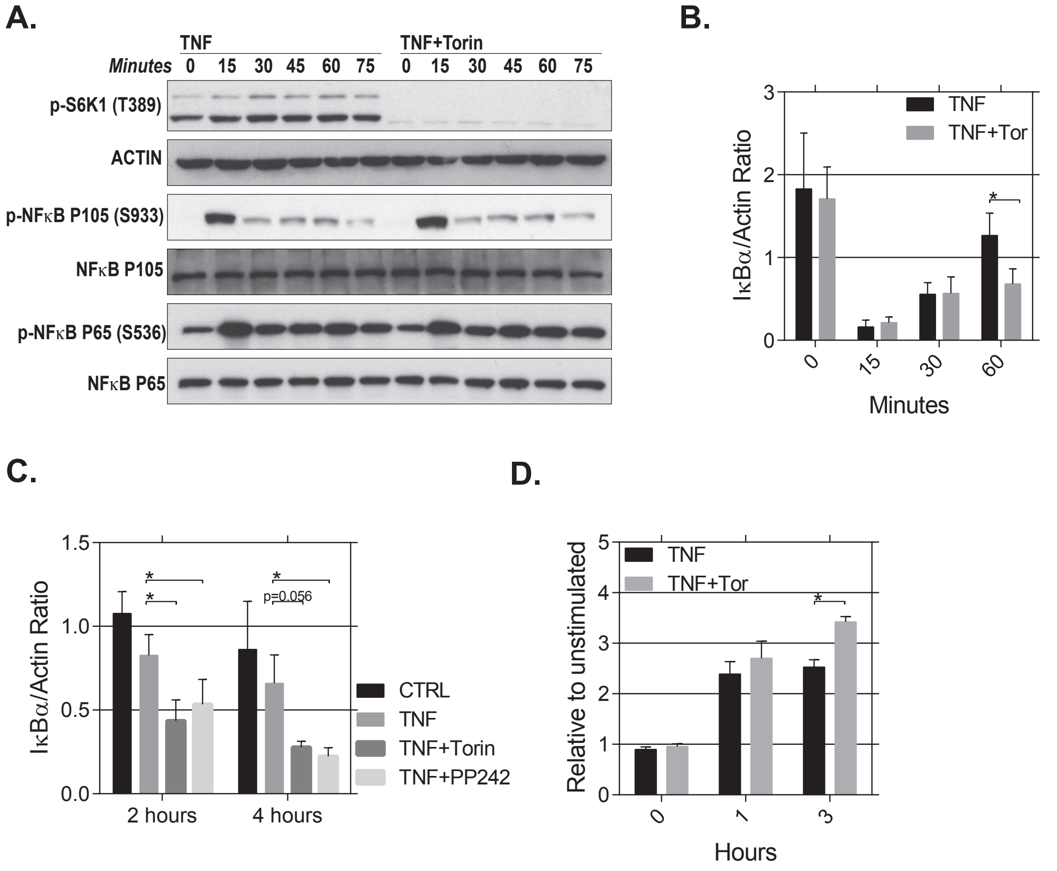

Figure S5. mTOR affects NF-κB signaling by influencing IκBα dynamics. Related to Figure 5.
A. Western blots of RA-FLS that were pretreated with either DMSO or Torin (250 nM) for 60 minutes and then stimulated with TNF (10 ng/ml) for indicated time periods. Representative blots of at least three independent experiments are shown. B. and C. Semiqunatitative analysis of IκB-α levels in TNF, TNF+Torin or TNF+PP242 treated RA-FLS. Immunoblot band intensities from 5 (B.) or 3 (C.) independent experiments were measured by ImageJ software. IκB-α intensity was normalized to Actin. Values are the mean±SEM, *p<0.05, Student´s paired t-test. D. NF-κB DNA-binding activity by EMSA in nuclear extracts from RA-FLS, which were treated with DMSO or Torin-1 (250 nM) one hour prior to TNF stimulation (10 ng/ml). Band intensities were measured by ImageJ sofware. Band intensity was normalized to untreated controls, Values are the mean±SEM, *p<0.05, Student´s paired t-test. Data from four RA-FLS cell lines from different donors were pooled.

Figure S6.

A.

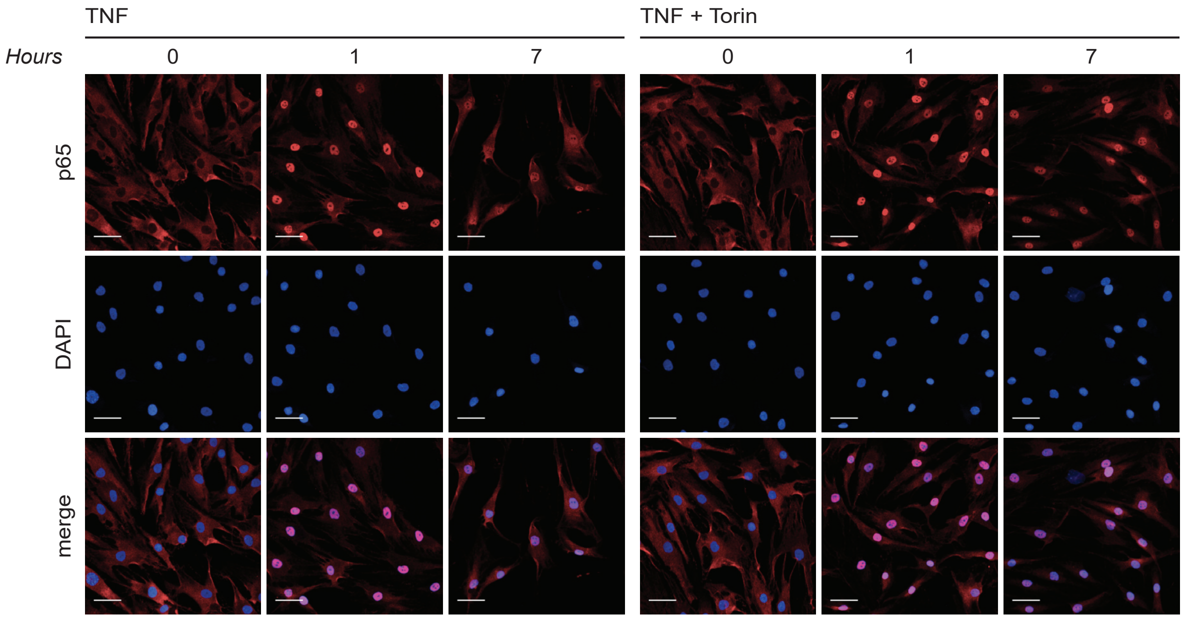

B.

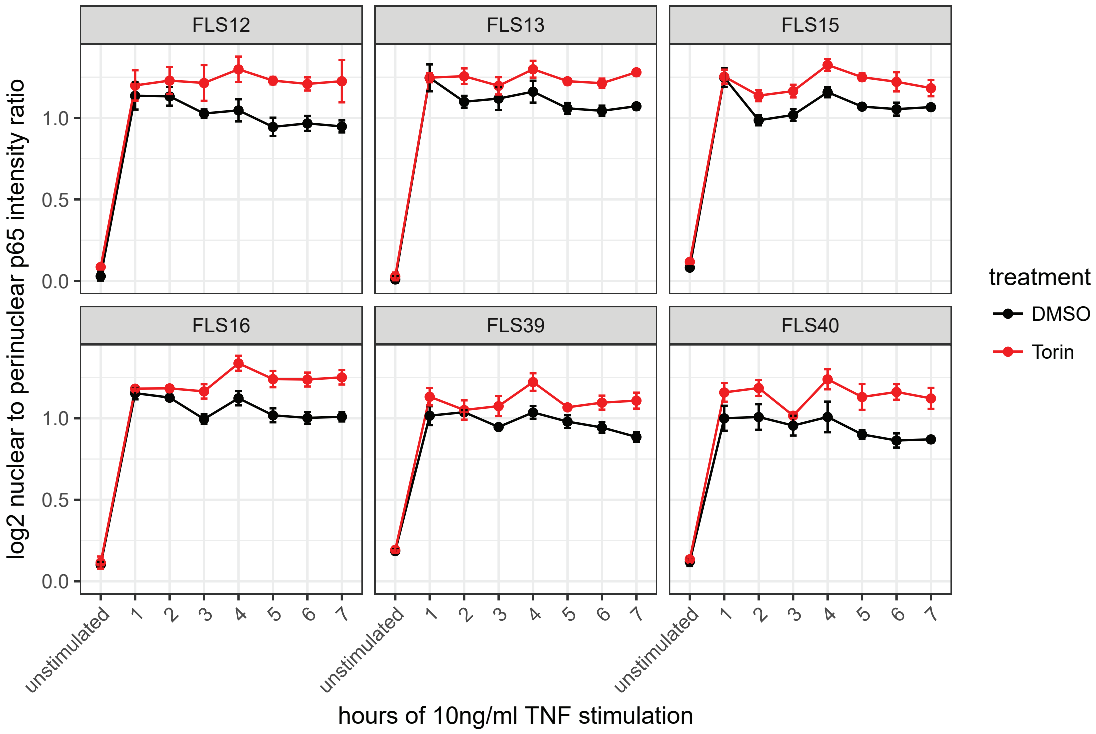

Figure S6. Related to Figure 5- mTOR affects NF-κB signaling by influencingIκBα dynamics. Related to Figure 5.
A. Immunofluorescence staining for P65, counterstaining with DAPI and merged images. Example images of automated imaging of RA-FLS, treated with either DMSO or 250nM Torin for 60 minutes prior to TNF stimulation. Scale bar represents 50µm. B. Log2 nuclear to perinuclear P65 signal ratios calculated from automated imaging and analysis for individual RA-FLS cell lines. Four technical replicates were performed.
